# Improving Microbiology Research: the Problems Are Less Statistical and More Biological

**DOI:** 10.1128/mBio.01634-16

**Published:** 2016-10-18

**Authors:** Dennis F. Lawler

**Affiliations:** Illinois State Museum, Springfield, Illinois, USA

## LETTER

I read with great interest the recent article by Casadevall et al. (1). The authors report outcomes of an American Academy of Microbiology colloquium that concluded by recommending six pathways to improving microbiology research. The recommendations are significantly removed from my expectations. As an experienced veterinarian, bioscience researcher, author, reviewer, and journal associate editor, my inner population clinician (my primary area) first recoiled at the disparity of our points of view. I have Dr. Casadevall to thank for directing the discussion toward some deeper issues.

In reality, many problems with bioscience research and publication are held more in common across disciplines than might be evident at first glance. For example, the most frequent research problems that I encounter are poor understanding of biological aspects of experimental design, misunderstanding of the basic biology of research subject species, and overinterpretation of study results. Thus, the errors are less statistical than they are biological and seem different from those reported by the Academy of Microbiology. But are they? One view of the contrast is that these are symptoms of a systemic dysfunction that is variable but nonetheless defining.

Consider several aspects of how we get to be scientists and ask if the process is misdirected. “Modern” undergraduate bioscience education has trivialized foundational training in zoological sciences, reducing these critical disciplines to components of other courses, if they are taught at all. Medical training of various types seems to produce good health care providers but instills little comprehension of research skills or ability to evaluate research publications. It is true enough that one does find very competent clinician-researcher folks but not at the frequency of past generations. Finally, as a scientific community, we clearly are training far more Ph.D. candidates than will be served by available employment, thus creating a competitive culture of sensationalism (a substitute term for taking shortcuts to the head of the line for research funding). For the latter assertion, there exists an unfortunate abundance of published support involving some supposedly “high-impact” journals that now seem more like supermarket science fiction. It is no longer clear what the impact factor impacts. Perhaps, it should be discarded and thus present temptation no longer.

The foundations of bioscience education seem to be quaking and cracking before our very eyes. Has the time come at length to focus more appropriately on solutions? Solutions can be evaluated and applied within disciplines, but if the underlying problems are systemic across disciplines, is that enough? Should we not take a thorough and introspective look at the entire process, beginning with undergraduate curricula and only then progressing into graduate and postgraduate education? Certainly, that is not a task for the faint of heart. Neither is it a single-discipline task.

The idea of One Health is not new, but its resurgence offers opportunity. We should observe that interprofessional research collaboration has become critically important to advancing knowledge and providing beneficial implementations thereof, across all aspects of health care, from the biochemistry laboratory to the clinic. Why? The scientific problems of today are very complex and multidimensional. So must be the solutions. Quoting the One Health website (http://www.onehealthcommission.org), “One Health is the collaborative effort of multiple health science professions, together with their related disciplines and institutions—working locally, nationally, and globally—to attain optimal health for people, domestic animals, wildlife, plants, and our environment.” Please note the part about “related disciplines and institutions”—that would be you, dear reader. The open-minded scientist in you is needed in bioscience and medicine.

I would like to call for a thoughtful, broad, and forceful effort to reform science education. We are training far too many bioscience students far too narrowly across disciplines, and most do not understand how to communicate outside the silos that are created thereby. The mess that is today’s scientific literature is all the evidence that we need to understand that it is time to tear down the silos and talk honestly among ourselves about real solutions.
